# Anesthetic Management for Valve-in-Valve Transcatheter Aortic Valve Replacement (TAVR) After Primary TAVR Failure With Mixed Aortic Valve Dysfunction

**DOI:** 10.7759/cureus.89552

**Published:** 2025-08-07

**Authors:** John Choi, Jennifer Shmukler, Derek He

**Affiliations:** 1 Adult Cardiothoracic Anesthesiology, Brigham and Women's Hospital, Boston, USA; 2 Anesthesiology, Mount Sinai Hospital, New York City, USA

**Keywords:** cerebral oximetry, general anesthesia, hemodynamic monitoring, rapid ventricular pacing, redo tavr, transesophageal echocardiography, valve-in-valve tavr, vasoactive support

## Abstract

Redo valve-in-valve transcatheter aortic valve replacement (TAVR) confronts anesthesiologists with compounded hemodynamic and neurologic risk. We managed an 85-year-old male with severe mixed prosthetic aortic dysfunction whose pre-procedural transthoracic echocardiogram showed a peak velocity of 3.7 m/s⁻¹, a mean gradient of 24 mm Hg, an effective orifice area of 0.8 cm², and a posteriorly directed, eccentric regurgitant jet filling >50% of the left ventricular outflow tract. The failure was suspected to have been caused by structural valve deterioration within five years after the initial TAVR. CT confirmed adequate coronary heights and true-lumen dimensions for a 26 mm Sapien 3 Ultra Resilia valve.

General anesthesia was induced with 20 mg of etomidate and 200 mcg of fentanyl and maintained with low-dose propofol (50-100 mcg/kg/min) and remifentanil (0.05-0.1 mcg/kg/min), permitting rapid titration around two rapid-ventricular-pacing (180 bpm) runs. Continuous transesophageal echocardiography (TEE)-guided balloon valvuloplasty, valve deployment, and real-time assessment of ventricular filling; anticipatory epinephrine infusion plus norepinephrine boluses countered pacing-induced hypotension. Cerebral oximetry monitored regional saturation during hemodynamic excursions.

Intra-procedural TEE confirmed coaxial positioning, full expansion of the new prosthesis, and immediate abolition of the eccentric regurgitant jet. Post-deployment deep-gastric views demonstrated a peak velocity of 2.3 ms⁻¹, trivial central regurgitation, and preserved biventricular function; aortic-root angiography corroborated the absence of coronary obstruction or paravalvular leak. The patient was extubated in the hybrid suite, required <2 h of low-dose norepinephrine (0.02-0.06 mcg/kg/min), ambulated on postoperative day one, and was discharged home on day two.

This case illustrates how detailed preoperative imaging, real-time TEE guidance, and proactive vasoactive strategies enable hemodynamic stability, neuroprotection, and fast-track recovery in high-risk redo TAVR.

## Introduction

Valve-in-valve transcatheter aortic valve replacement (TAVR) has become a critical intervention for patients whose initial TAVR fails due to structural valve deterioration, malposition, or mixed stenosis and regurgitation, offering a less invasive alternative to surgical explantation in high-risk populations [1]. Although redo transcatheter aortic valve replacement (TAVR) comprises only 0.5-0.7% of all TAVR procedures, contemporary multicenter registries report technical success rates exceeding 90%, with mid-term survival comparable to that of first-time TAVR but with distinct procedural risks such as coronary obstruction and patient-prosthesis mismatch [1,2].

As the indications for TAVR broaden to include lower-risk and younger patients, anesthetic practice has gradually shifted from routine general anesthesia (GA) to monitored anesthesia care (MAC) with conscious sedation in straightforward cases [3]. However, complex ViV redo procedures, particularly in elderly patients with mixed aortic stenosis and regurgitation, frequently necessitate GA augmented by transesophageal echocardiography (TEE) and advanced hemodynamic monitoring to promptly detect and manage rapid hemodynamic swings during induction, rapid ventricular pacing, and valve deployment [4]. Valve-in-valve transcatheter aortic valve replacement (ViV TAVR) has become a critical intervention for patients whose initial TAVR fails due to structural valve deterioration, malposition, or mixed stenosis and regurgitation, offering a less invasive alternative to surgical explantation in high-risk populations [1]. Although redo TAVR comprises only 0.5-0.7% of all TAVR procedures, contemporary multicenter registries report technical success rates exceeding 90%, with mid-term survival comparable to that of first-time TAVR but with distinct procedural risks such as coronary obstruction and patient-prosthesis mismatch [1,2].

As the indications for TAVR broaden to include lower-risk and younger patients, anesthetic practice has gradually shifted from routine general anesthesia (GA) to monitored anesthesia care (MAC) with conscious sedation in straightforward cases [3]. However, complex ViV redo procedures, particularly in elderly patients with mixed aortic stenosis and regurgitation, frequently necessitate GA augmented by transesophageal echocardiography (TEE) and advanced hemodynamic monitoring to promptly detect and manage rapid hemodynamic swings during induction, rapid ventricular pacing, and valve deployment [4].

Optimal anesthetic management hinges on the selection of induction agents with minimal cardiovascular depression (e.g., etomidate, fentanyl), proactive vasopressor strategies during pacing, and continuous TEE to assess ventricular function and valve position in real time [3,5]. Tailoring these approaches to patients with limited cardiac reserve and multiple comorbidities is essential to minimize perioperative morbidity and mortality.

In this report, we detail the perioperative anesthetic considerations and approaches employed for an 85-year-old man undergoing ViV TAVR after primary TAVR failure resulting in severe mixed aortic stenosis and insufficiency, with the aim of guiding anesthesiologists managing similar high-risk scenarios. Optimal anesthetic management hinges on the selection of induction agents with minimal cardiovascular depression (e.g., etomidate, fentanyl), proactive vasopressor strategies during pacing, and continuous TEE to assess ventricular function and valve position in real time [3,5]. Tailoring these approaches to patients with limited cardiac reserve and multiple comorbidities is essential to minimize perioperative morbidity and mortality.

In this report, we detail the perioperative anesthetic considerations and approaches employed for an 85-year-old man undergoing ViV TAVR after primary TAVR failure resulting in severe mixed aortic stenosis and insufficiency, with the aim of guiding anesthesiologists managing similar high-risk scenarios.

## Case presentation

**Figure 1:**
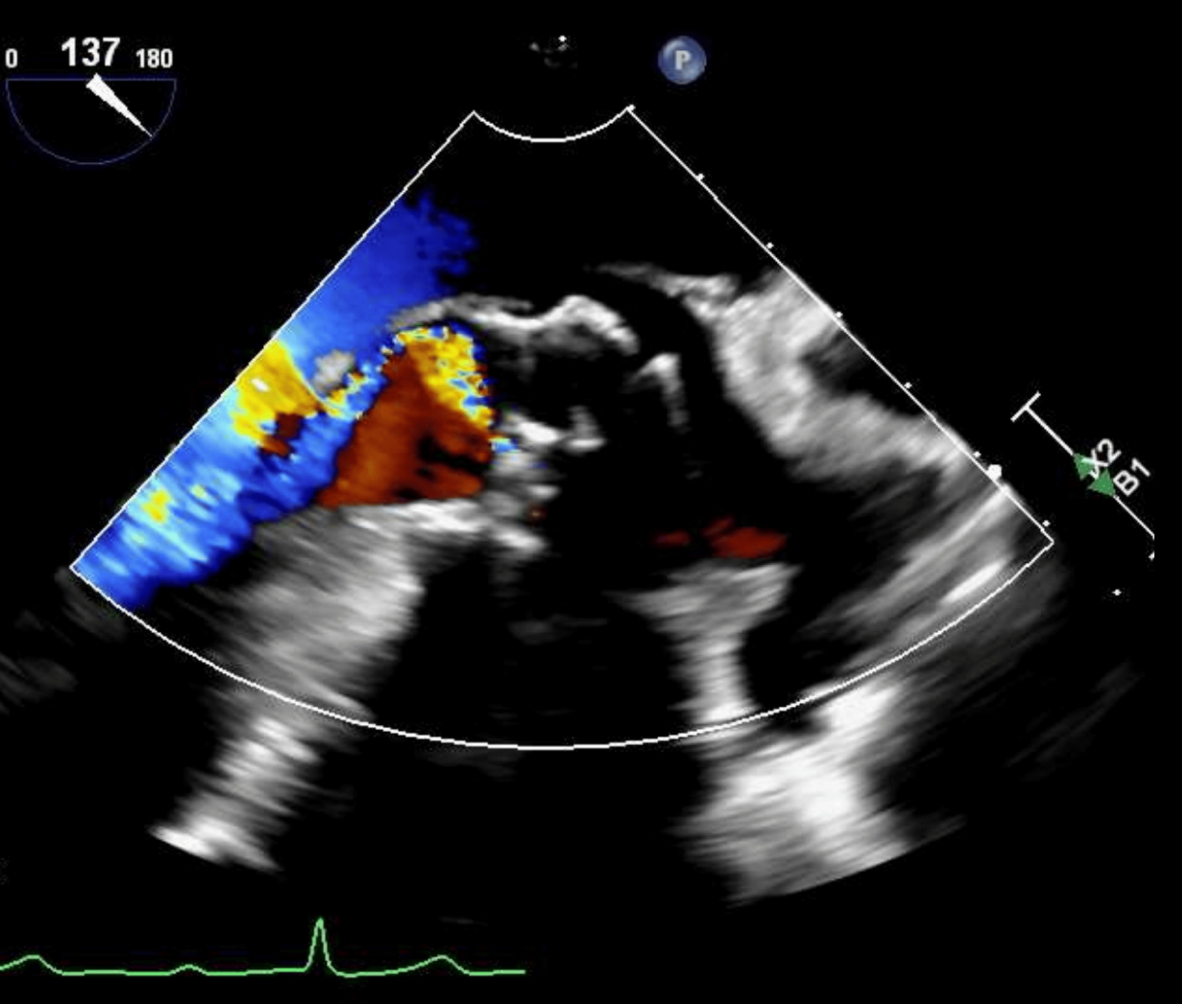
TEE in midesophageal long axis view showing moderate to severe aortic insufficiency with turbulent, transvalvular posteriorly directed eccentric jet.

**Figure 2:**
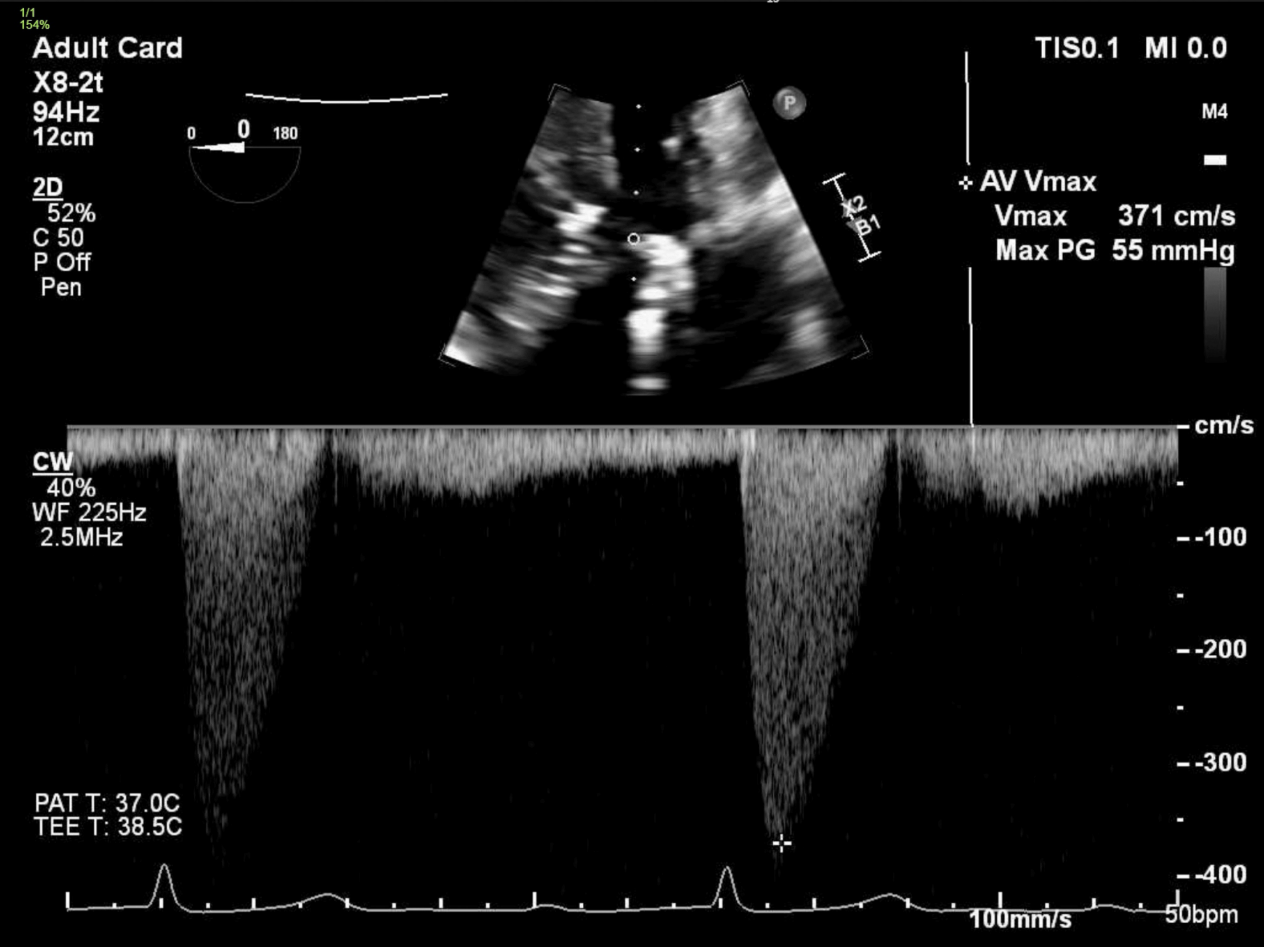
TEE in deep gastric view showing peak anterograde velocity through aortic valve of 3.7 m/s, consistent with moderate aortic stenosis.

An 85-year-old man with a history of hypertension, hyperlipidemia, type 2 diabetes mellitus (A1c 6.5%), heart failure with preserved ejection fraction (LVEF 50% in August 2024), three-vessel coronary artery disease, chronic lacunar strokes, proximal basilar artery stenosis, subacute subdural hematoma one month prior, and prior TAVR (Edwards S3 26 mm in 2019) presented with progressive dyspnea on exertion and fatigue. Transthoracic echocardiography revealed moderate transvalvular aortic stenosis with moderate regurgitation (Figures 1, 2). Given his age, comorbidities, and an STS-PROM of 3.0% for surgical aortic valve replacement (AVR), he was deemed a poor surgical candidate. He underwent several days of medical optimization, primarily aggressive diuresis, to relieve volume overload and improve respiratory symptoms before proceeding to valve-in-valve TAVR.

In the hybrid operating room, standard ASA monitors were applied, and a 20-gauge left radial arterial line was placed for beat-to-beat hemodynamic monitoring. A single 14-gauge peripheral IV was established in the left antecubital fossa. After preoxygenation, general endotracheal anesthesia was induced, and the trachea was secured with an 8.0 mm cuffed endotracheal tube. Following intubation, the interventional team placed a 7 Fr femoral venous sheath in the right groin. A bispectral index (BIS) sensor was applied to guide anesthetic depth. General anesthesia was selected because complex ViV deployment required continuous TEE guidance, controlled ventilation for precise valve positioning, and immediate hemodynamic rescue capability during prolonged rapid-pacing runs, which are needs that conscious sedation could not reliably meet in this frail patient.

Anesthesia was maintained with propofol (50-100 µg/kg/min) and remifentanil (0.05-0.2 µg/kg/min) infusions for rapid titration. Norepinephrine and epinephrine infusions were prepared; norepinephrine was titrated to maintain systolic blood pressure at the patient’s baseline (≈140 mm Hg), given his extensive coronary artery disease. Epinephrine infusion was initiated during rapid pacing runs to support cardiac output, and intermittent norepinephrine boluses were administered to counteract pacing-induced hypotension.

Transesophageal echocardiography provided continuous assessment of ventricular function and valve deployment. After systemic heparinization with a heparin dose of 80 IU/kg and ACT > 250 s, rapid ventricular pacing at 180 bpm was first employed during balloon valvuloplasty of the failed Sapien 3 frame to minimize movement. A second pacing run then facilitated deployment of the 26 mm Sapien 3 Ultra Resilia valve within the prior prosthesis. Throughout both balloon and valve deployment runs, epinephrine infusion and norepinephrine boluses maintained hemodynamic stability. Post-deployment TEE demonstrated coaxial alignment, full frame expansion, normal leaflet excursion, no thrombus, and trivial central regurgitation without paravalvular leak.

**Figure 3:**
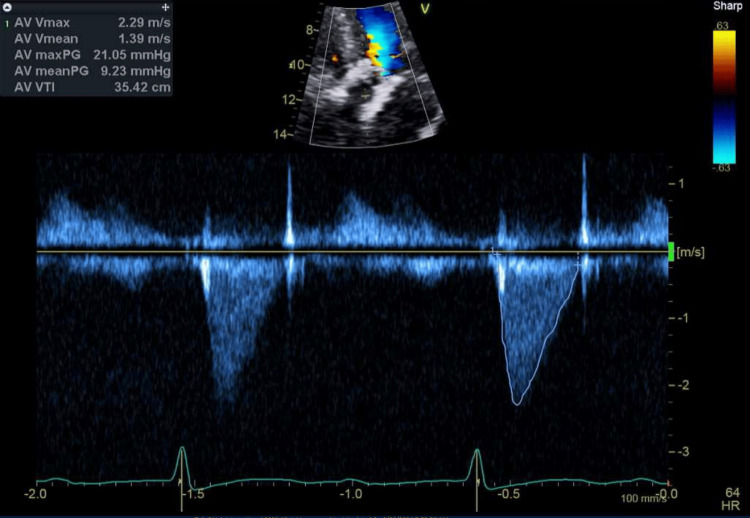
TEE in deep gastric view showing peak transaortic velocity of 2.3 m/sec, consistent with mild aortic stenosis and improved hemodynamics.

Following successful valve deployment and confirmation of position and function by TEE and aortic root angiography (no paravalvular leak), all sheaths were removed and percutaneous closure achieved. Total procedure time was 110 min (skin-to-skin), fluoroscopy time 14 min, and contrast volume 82 mL of iso-osmolar iodixanol. The patient emerged smoothly from anesthesia, was extubated in the hybrid suite, and was transferred to the cardiac intensive care unit on minimal norepinephrine support. He required no further vasoactive support and was mobilized on postoperative day one. A follow-up echocardiogram confirmed stable valve function, with the new valve demonstrating good leaflet mobility and central flow with mild central transvalvular regurgitation, with measurements consistent with excellent hemodynamic results (Figure 3). The patient was discharged home on postoperative day two in stable condition.

## Discussion

Redo ViV TAVR presents unique anesthetic challenges due to the combination of advanced age, multiple comorbidities, and mixed aortic valve dysfunction. While monitored anesthesia care with conscious sedation is increasingly employed for primary TAVR in low-risk patients, complex ViV procedures, especially in octogenarians with limited cardiac reserve, often warrant general anesthesia (GA) to facilitate transesophageal echocardiography (TEE) guidance and rapid intervention for hemodynamic instability [6]. Comparative studies suggest that GA may be associated with longer procedural times but affords greater control over airway and hemodynamics, potentially improving safety during rapid ventricular pacing and valve deployment [6,7].

Rapid ventricular pacing (RVP) is integral to both balloon valvuloplasty and valve deployment, as it transiently reduces cardiac output to minimize prosthesis migration. However, RVP induces abrupt hypotension that must be anticipated and managed proactively. In our case, an epinephrine infusion during pacing runs, supplemented by intermittent norepinephrine boluses, stabilized systemic pressures and maintained coronary perfusion in the setting of three-vessel disease [8]. Continuous TEE monitoring allowed real-time assessment of ventricular function and immediate detection of paravalvular leak, guiding fluid and vasoactive therapy [9].

Moreover, implementation of cerebral oximetry monitoring provided additional safety by detecting regional desaturations during hypotensive episodes, which was particularly valuable given the patient’s history of cerebrovascular disease and basilar artery stenosis [10]. The strategy of early extubation and fast-track recovery aligns with enhanced recovery protocols in TAVR and may contribute to shorter ICU stays and earlier mobilization without increasing respiratory complications [11].

Future research should aim to define optimal anesthetic protocols for ViV TAVR, balancing sedation depth, hemodynamic stability, and neuroprotection in patients with high cerebrovascular risk. Multidisciplinary planning remains essential to anticipate procedural risks and streamline perioperative care for these complex cases.

## Conclusions

In conclusion, this case shows that redo valve-in-valve TAVR in a frail octogenarian can be completed safely when anesthesia is tightly integrated with detailed pre-procedural imaging. Transthoracic echocardiography and multidetector CT clarified valve sizing, coronary clearance, and procedural risks, while continuous intra-operative TEE and cerebral oximetry guided real-time decisions. An induction sequence using etomidate and fentanyl, followed by low-dose propofol and remifentanil, minimized myocardial depression; baseline norepinephrine with epinephrine supplementation during rapid-pacing runs preserved coronary and cerebral perfusion. These measures maintained hemodynamic stability, allowed extubation in the hybrid suite, reduced postoperative vasoactive requirements, and supported discharge on postoperative day two. Clinicians facing similar redo TAVR cases should emphasize comprehensive imaging, cardiovascularly gentle induction, anticipatory vasoactive therapy timed to pacing, real-time TEE surveillance, and multimodal neuro-monitoring to optimize safety and promote fast-track recovery.

## References

[1] Sava RI, Garot P, Benamer H (2025). Redo-transcatheter aortic valve replacement procedural optimization and patient selection: from bench to clinical practice. J Clin Med.

[2] Gallo M, Dvir D, Demertzis S (2016). Transcatheter valve-in-valve implantation for degenerated bioprosthetic aortic and mitral valves. Expert Rev Med Devices.

[3] Hayanga HK, Woods KE, Thibault DP (2023). Anesthetic management for transcatheter aortic valve replacement: a national anesthesia clinical outcomes registry analysis. Ann Card Anaesth.

[4] Abbett SK, Urman RD, Resor CD, Brovman EY (2021). The effect of anesthesia type on outcomes in patients undergoing transcatheter aortic valve replacement. J Cardiothorac Vasc Anesth.

[5] Kherallah RY, Koneru S, Krajcer Z (2021). Hemodynamic outcomes after valve-in-valve transcatheter aortic valve replacement: a single-center experience. Ann Cardiothorac Surg.

[6] Holmes HR, Falasa M, Neal D (2022). Monitored anesthesia care versus general anesthesia for transcatheter aortic valve replacement. Innovations (Phila).

[7] Kodali S, Thourani VH, White J (2016). Early clinical and echocardiographic outcomes after SAPIEN 3 transcatheter aortic valve replacement in inoperable, high-risk and intermediate-risk patients with aortic stenosis. Eur Heart J.

[8] Fadah K, Khalafi S, Corey M (2024). Optimizing anesthetic selection in transcatheter aortic valve replacement: striking a delicate balance between efficacy and minimal intervention. Cardiol Res Pract.

[9] Pibarot P, Hahn RT, Weissman NJ, Monaghan MJ (2015). Assessment of paravalvular regurgitation following TAVR: a proposal of unifying grading scheme. JACC Cardiovasc Imaging.

[10] Fanning JP, Walters DL, Wesley AJ (2017). Intraoperative cerebral perfusion disturbances during transcatheter aortic valve replacement. Ann Thorac Surg.

[11] Smith CR, Leon MB, Mack MJ (2011). Transcatheter versus surgical aortic-valve replacement in high-risk patients. N Engl J Med.

